# Editorial: Innovative tools to support the elimination of neglected tropical diseases (NTDs)

**DOI:** 10.3389/fmicb.2023.1208113

**Published:** 2023-05-10

**Authors:** Wei Wang, Jian Li, Mohamed R. Habib

**Affiliations:** ^1^National Health Commission Key Laboratory of Parasitic Disease Prevention and Control, Jiangsu Provincial Key Laboratory of Parasite and Vector Control Technology, Jiangsu Institute of Parasitic Diseases, Wuxi, Jiangsu, China; ^2^Department of Human Parasitology, School of Basic Medical Science, Hubei University of Medicine, Shiyan, Hubei, China; ^3^Medical Malacology Department, Theodor Bilharz Research Institute, Giza, Egypt

**Keywords:** neglected tropical diseases (NTDs), diagnosis, epidemiology, vaccine, elimination, immunology

Neglected tropical diseases (NTDs) are a heterogeneous group of 20 diseases of parasitic, bacterial, viral, fungal, and non-communicable origin that affect approximately one billion impoverished communities around the world. The global age-standardized incidence and number of incident NTDs cases increased between 1990 and 2019, indicating that NTDs remain a substantial public health problem worldwide (Lin et al., 2022). Urged by two road maps for the prevention, control and elimination of NTDs by the World Health Organization (WHO, 2012; Malecela and Ducker, 2021), significant progress has been made in the management of NTDs. The current Research Topic consists of 13 publications covering biology, immunology, epidemiology, diagnosis, and vaccines for NTDs, with the goal of introducing innovative tools implemented to support global elimination of NTDs and facilitating translation of these tools into disease-affected regions.

Epidemiological studies are critical for developing and evaluating disease control strategies, as well as serving as a guide for the management of patients who have already developed illness (Bannister-Tyrrell and Meiqari, 2020). Visceral leishmaniasis has recently reemerged in China (Zheng et al., 2020). Based on data captured from the Infectious Disease Reporting Information Management System of the Chinese Center for Disease Control and Prevention, Li et al. investigated the epidemiological features and spatial-temporal clusters of visceral leishmaniasis in mainland China from 2019 to 2021. The authors identified mountain-type zoonotic visceral leishmaniasis as the predominant type, with peasants and infants as the dominant high-risk populations. The highest peak incidence was recorded between March and May, with 69.57% of cases representing individuals aged 15 years and older. In addition, spatial clustering analysis demonstrated the high-incidence areas of visceral leishmaniasis had shifted to central China, notably in Shanxi and Shaanxi provinces. In the present Research Topic, Yao et al. explored the epidemiology and genetic diversity of the Gram-negative bacterium *Bartonella* spp., the causative agent of bartonellosis in mammals (Chomel and Kasten, 2010). The authors reported a 6.4% prevalence rate of *Bartonella* in rodents in Guangzhou, southern China, in 2020. Molecular analyses based on the *rrs, gltA*, and *rpoB* genes characterized six *Bartonella* species in captured rodents, including *B. queenslandensis, B. mastomydis, B. tribocorum, B. rattimassiliensis, Bartonella* sp. AA86HXZ, and *Bartonella* sp. Fuji 12-1.

Ticks are regarded as the second most important vectors of human disease in the world after mosquitoes (de la Fuente et al., 2008). One of the infections carried by ticks is *Anaplasma* spp., the causative agent of anaplasmosis. It is a severe threat to public health and causes huge economic losses on cattle farms. Qi et al. investigated the co-infections and co-existence of several *Anaplasma* spp. and variants in hedgehogs and ticks feeding on hedgehogs and cattle collected in Jiangsu province, Eastern China. They discovered important pathogenic *Anaplasma* spp., such as *A. phagocytophilum, A. marginale*, three *A. platys* variants, and two *A. bovis* variants, as well as a novel Candidatus *Cryptoplasma* sp., with varying prevalence in ticks and hedgehogs, and different co-existence combinations were observed only in ticks rather than hedgehogs. Hedgehogs may be more important reservoirs for *A. bovis* than *A. platys*, and *Anaplasma* spp. may be transmitted horizontally between tick species via common hosts. In the current study, the coexistence of several *Anaplasma* spp. or variants may accelerate the creation of novel variants, represent significant hazards to public health as well as economic losses from animal farming, and raise detection and diagnosis issues in the investigated area. With the identification of key pathogenic *Anaplasma* spp. and their co-infections/co-existence in ticks, the current study presented epidemiological data that could be critical for developing strategies for early warning, prevention, and management of possible *Anaplasma*. spp. infections. In addition, Zhang et al. detected a 2.53% prevalence rate of Meihua Mountain virus, a novel member of Nairobi sheep disease orthonairovirus, in ticks captured from cattle and wild boars in Fujian province, Southeastern China from 2019 to 2020.

NTDs control efforts require precise diagnosis (Souza et al., 2021). Jiao et al. developed a convenient, rapid, specific, and sensitive assay combining recombinase-aided amplification and lateral flow strip assay (RAA-LFA) that targeted the *CPSIT_RS02830* gene of *Chlamydia psittaci* for detection of active *C. psittaci* infection, and the RAA-LFA, which was completed within 15 min at a single temperature of 39°C, showed a diagnostic sensitivity of 1 × 100 copies/μl *C. psittaci* DNA, and no cross-reactivity with DNA samples from other intracellular pathogens. In addition, this RAA-LFA tested positive for *C. psittaci* in all fecal samples from mice infected with *C. psittaci* 1 day post-infection.

Based on four helper T lymphocyte (HTL) epitopes, five cytotoxic T lymphocyte (CTL) epitopes and three B-cell epitopes predicted from five patent tuberculosis infections and *Mycobacterium tuberculosis* region of difference (LTBI-RD)-related antigens, Cheng et al. constructed C543P, a novel polypeptide molecule (PPM), which presented high antigenicity, immunogenicity and stability. This C543P candidate was found to activate T and B lymphocytes, and produce high levels of Th1 cytokines, suggesting that C543P may be a promising biomarker for the diagnosis of latent tuberculosis infection (LTBI). Additionally, Zhong et al. reviews the diagnostic potential of microRNAs in schistosomiasis and Quansah et al. reviews the value of the CRISPR-Cas13 system for diagnosis of malaria.

The interaction between the host, indigenous microbiota, and pathogens has been reported to alter the outcome of infections (Libertucci and Young, 2019). Guan et al. found that *Plasmodium yoelii* infection altered the gut microbiota composition in mice, and Zhao et al. reported that the altered microbiota of *Aedes albopictus* affected its susceptibility to dengue fever virus. Alveolar echinococcosis, caused by the larval stage of the tapeworm *Echinococcus multilocularis*, is known as “parasitic cancer” due to its mortality (Xu and Ahan, 2020). Feng et al. found that the complex of key cell cycle regulators EmCyclinD and EmCDK4/6 contributed to the cell cycle regulation of germinative cells in *E. multilocularis* via the EGFR-ERK-EmCyclinD pathway.

Vaccination is widely acknowledged to be one of the most cost-effective health interventions for both human and animal populations (Ghattas et al., 2021). Liang et al. generated an *L. ivanovii* hemolysin gene deletion strain LIΔ*ilo* and a modified strain LIΔ*ilo:hly* of *L. ivanovii*, and the recombinant strain LIΔ*ilo:hly* was found to exhibit high biosafety and immunogenicity, making it a suitable vector for vaccine production. In another review article, Tang et al. discussed the several types of vaccines available for trichinellosis, a worldwide food-borne zoonotic NTD, examined the factors influencing immunization effectiveness, and provided views and difficulties for future vaccine development.

In conclusion, this Research Topic offers unprecedented insights into and innovative strategies for the management of NTDs. We expect that the topic will help accelerate efforts toward the global elimination of NTDs.

## Author contributions

All authors listed have made a substantial, direct, and intellectual contribution to the work and approved it for publication.
